# Development of a balanced instrument to measure global health-related quality of life: The 13-MD

**DOI:** 10.3389/fpsyt.2022.837510

**Published:** 2022-09-06

**Authors:** Moustapha Touré, Alain Lesage, Thomas G. Poder

**Affiliations:** ^1^Department of Economics, Business School, Université de Sherbrooke, Sherbrooke, QC, Canada; ^2^Centre de Recherche de l'IUSMM, CIUSSS de l'Est de L'île de Montréal, Montréal, QC, Canada; ^3^Department of Psychiatry, Faculty of Medicine, Université de Montréal, Montréal, QC, Canada; ^4^Department of Management, Evaluation and Health Policy, School of Public Health, Université de Montréal, Montréal, QC, Canada

**Keywords:** global health, economic assessment, health-related quality of life (HRQoL), quality-adjust life year (QALY), generic instrument

## Abstract

Generic instruments are of interest in measuring global health-related quality of life (GHRQoL). Their applicability to all patients, regardless of their health profile, allows program comparisons of whether the patients share the same disease or not. In this setting, quality-adjusted life-year (QALY) instruments must consider GHRQoL to allow the best programs to emerge for more efficiency in health resource utilization. However, many differences may be perceived among the existing generic instruments relative to their composition, where dimensions related to physical aspects of health are generally depicted more often than dimensions related to mental or social aspects. The objective of this study was to develop a generic instrument that would be complete in its covered meta-dimensions and reflect, in a balanced way, the important aspects of GHRQoL. To this end, a Delphi procedure was held in four rounds, gathering 18 participants, including seven patients, six caregivers, and five citizens. The structure of the instrument derived from the Delphi procedure was submitted to psychometric tests using data from an online survey involving the general population of Quebec, Canada (*n* = 2,273). The resulting questionnaire, the 13-MD, showed satisfying psychometric properties. It comprises 33 items or dimensions, with five to seven levels each. The 13-MD reflects, in a balanced form, the essential aspects of GHRQoL by including five meta-dimensions for physical health, four meta-dimensions for mental health, three meta-dimensions for social health, and one meta-dimension for sexuality and intimacy. The next step will involve the development of a value set for the 13-MD to allow QALY calculation.

## Introduction

When health technologies are subject to increasing improvements, health systems face a continuously increasing demand for health services, in contrast with resource scarcity. The principle of efficiency in resource utilization implies that choices should be made to allow the more impactful technologies on people's health to emerge at a lower cost. In this way, over the years, the quality-adjusted life-year (QALY) became one of the main instruments used to measure the benefit in the economic evaluation of health programs and technologies worldwide (1). The principle of QALY is to combine the duration (mortality) and quality (morbidity) of life into a single measure (2). This combination allows us to consider the patients' perceived health-related quality of life (HRQoL) to capture the comparative effectiveness of health care interventions or programs (3). The Q in QALY, or utility in health, is a concept that defines the wellbeing or satisfaction level that a person gets from being in a given health state (4). Many QALY instruments have been developed for this purpose. These instruments help to determine patient health utility over a period of 1 year, which is between zero and one, where one is the state of perfect health and zero is the state of death. In the patient's perception, it is possible to have health states worse than death; in those cases, a negative value is assigned. It is necessary to distinguish between specific and generic instruments (5). Specific instruments are more oriented toward specific conditions or diseases but do not allow HRQoL comparison between patients with different diseases. Meanwhile, generic instruments are adapted for all types of health profiles to allow for the comparison between patients with different diseases (6). However, many differences may be perceived among the generic instruments available regarding their composition, where dimensions related to physical aspects of health are depicted more often than dimensions related to mental or social aspects (7). The general observation is that almost all generic instruments contain one or more dimensions related to physical aspects (e.g., disability, discomfort/pain) at the expense of other dimensions, such as mental/cognitive function, wellbeing/happiness, or sadness/depression (7). Additionally, few instruments consider dimensions relative to the social domain (7). Thus, clear differences are noticed in generic instruments that aim to measure the patient's perceived global health-related quality of life (GHRQoL) most effectively. These instruments seem to measure different things, and to date, there is no consensus on the best instrument to measure the Q in QALY (6, 8). For example, Richardson et al. (9) found that an individual with visual and hearing impairments had a utility score of 0.14 if the instrument used was the Health Utility Index (HUI) 3 compared to a score of 0.8 if the instrument used was the EuroQol 5-Dimension (EQ-5D). Thus, a program allowing this individual to return to a state of perfect health (utility score of one) would have recorded a 4.3 times greater gain in utility using the HUI-3 rather than the EQ-5D. Therefore, for program choice purposes, using the EQ-5D would mean that this program would have the same effect on the cost/QALY ratio as the 4.3-fold increase in cost. This example, which may seem excessive, relates to two frequently used instruments and demonstrates the potential differences caused by the choice of one or the other instrument. As these differences are strongly related to the dimensions covered by the instruments, there is a need to have a generic instrument that would be more complete in the dimensions covered to measure GHRQoL.

According to the World Health Organization (WHO), health is “a state of complete physical, mental, and social wellbeing and not merely the absence of disease or infirmity” (10–13). This definition considers all health dimensions to be critical and fundamental in one's life. Consequently, they all must be considered in a balanced way in health care decision-making. To do so, instruments meant to measure people's GHRQoL must allow all dimensions (i.e., physical, mental, and social health) to be adequately represented. In this setting, we developed a new instrument to measure GHRQoL with the aim of being more complete and more balanced. To do so, a Delphi procedure allowed citizens, patients, and caregivers to share their opinions on the dimensions to be included in the new instrument. After the Delphi procedure, which was conducted in several rounds, the instrument was tested on a large sample of the general population for psychometric testing. This article suggests a new generic instrument, the 13-MD, which comprises 33 items or dimensions with five to seven levels each (i.e., a level corresponding to a response option). This study thus presents the process of development of a new GHRQoL instrument for which a value set that will be developed in the near future will allow us to use it as a QALY instrument for cost-utility analysis.

## Methods

### Delphi procedure

A Delphi procedure was used for the creation of the new generic instrument. It is an iterative procedure that allows the collection of viewpoints on a specific subject using surveys with feedback (14). Several rounds may be required for the Delphi; in each round, a new questionnaire based on the results of the previous round is made available. The questionnaires focus on specific matters, opportunities, or solutions. The procedure ends when the research issue finds an answer; that is, when a consensus or the theoretical saturation threshold is reached or when we have enough information about the topic (14). In this study, conducted during the COVID-19 pandemic, an online survey was preferred, including patients, caregivers, and citizens; uniformity in different categories' sample sizes were sought. The anonymity of participants was guaranteed during all procedures so that participants could fully express their opinions. In that regard, before the beginning of the study, every participant was assigned a unique code to be confidentially identified by the research team. The analysis of the results reveals that subjects were treated as a group. Patients were recruited with the help of the Patients' committee of the CIUSSS de l'Est de l'Île de Montréal, Montreal, Canada, while caregivers were recruited with the help of the recovery mentoring microprogram offered in the Faculty of Medicine of the University of Montreal. For citizens, researchers' contacts were solicited through word of mouth, as were already recruited participants' relatives.

There is no consensus in the literature on the number of participants to be included in a Delphi study. Diverse sample sizes are proposed by authors, but some generally established rules of thumb indicate a range between 15 and 30 participants (15, 16). According to this convention, we sought 18 participants to compose the study sample, and a representative sample in terms of age (18–80 years), gender (1:1), and education level were requested. Nevertheless, considering that people over 40 years old would possibly present more physical or mental comorbidities, this population was privileged in the recruitment process. The 18 participants were then divided into six patients, six caregivers, and six citizens.

A solicitation e-mail was sent to each possible participant to ask for participation in the study. A positive response to the first e-mail was mandatory to receive the next e-mail with a consent form to be completed and returned. After this, the questionnaire's weblink was shared with the participants to begin the study. Completing the online survey was also considered a reaffirmation of consent to participate in the study. The survey platform LimeSurvey was used for the Delphi procedure.

For every concern or question, the participants could reach the person in charge of the study (i.e., the corresponding author) *via* the phone number provided in the letter of invitation and consent form or by responding to the email address provided in every email correspondence.

The objectives of the Delphi were to adjudicate the relevance of the proposed dimensions, modify them if needed, propose severity levels (i.e., response options that follow a gradation in the health description) for each dimension included, and propose new dimensions if necessary. A consensual approach was used to select the most important dimensions for the measurement of the new GHRQoL instrument. Three to four rounds were expected to be sufficient for the Delphi procedure. In each round, using specific questions, points of view, suggestions, and misunderstandings of respondents were collected anonymously (all respondents did not know the individual opinions of other participants but were informed of the global distribution of responses). In each round, a revised version of the questionnaire, based on the respondents' observations and suggestions in the previous round, was created—except for the first round—where the dimensions proposed were derived from the study by Touré et al. (7), which is a systematic review of the dimensions covered by HRQoL instruments and the different steps necessary for the creation of those instruments for QALY calculation. Further details on the selected studies and the dimensions can be found elsewhere (7).

In each round, specific questions were asked to judge the agreement level with the dimensions and levels. An average response time of 1 week was allocated to respondents after the online questionnaire was available. The procedure ended when a strong consensus was reached; that is, an agreement above 80% (17, 18).

### Psychometric validation

The dimensions determined to be the most important by the participants in the Delphi procedure underwent psychometric tests to establish the final dimensions and levels that would characterize the new generic instrument. To do so, an online survey was initiated with the general French-speaking population in Quebec, where the instrument derived from the Delphi was presented to them to assess its properties. A minimum of 1,702 participants were enrolled in the study to be representative of the general adult population with a confidence level of 90% and a margin of error of 2%. Participants were recruited in another study, where the questionnaire with the dimensions selected in the Delphi procedure was added. Participants were recruited by Dynata Inc. using a quota sampling method for gender, age, and education. The data collected went through a screening process that eliminated all incomplete observations. The scores used for the psychometrics analysis went from 1 (best level) to 5-7 (worst level). However, this is not the final scoring system since a value set is to be developed.

Internal reliability or consistency (i.e., the extent to which items are intercorrelated and measure the same construct) was assessed. To do so, Cronbach's alpha was calculated, and a value between 0.70 and 0.95 was targeted (19, 20). Items whose removal would allow a 2% rise in the Cronbach's alpha score were also targeted (19). The item-test correlation, measuring the item and the overall test correlation; the item-rest correlation, determining the item vs. the other items' correlation; and the inter-item correlation, analyzing the degree of correlation between items, were also calculated. We expected item-test coefficients to be high and roughly the same across items, item-rest coefficients to be >0.2 (21), and inter-item coefficients to be between 0.2 and 0.4 (22). Floor and ceiling effects, which represent the number of respondents that chose the worst and best possible answer, respectively (i.e., the worst and best levels describing health), were also checked in all items. This was done to ensure that the responses' levels allowed us to capture changes between users and that the instrument's reliability was not reduced (23). According to Terwee et al. (23), under 15% of responses must represent the best/worst score possible to exclude the absence of ceiling or floor effects.

The Bartlett test of sphericity and the Kaiser–Meyer–Olkin (KMO) test were also performed to establish the feasibility of performing an exploratory factor analysis (EFA) through a principal component analysis (PCA) (24) with varimax (orthogonal) rotation. The PCA would then help in observing the loadings' patterns and determining which items should be gathered in the same meta-dimension (i.e., groups containing items measuring a certain health aspect and are related) (3). Items explaining <20% of the components were seen as problematic.

The discriminant capacity (i.e., the degree to which the item discriminates between persons in different regions on the latent continuum) of the items was tested with the item response theory (IRT) model. IRT tries to explain/predict the relationship between latent traits (unobservable characteristics or attributes) and their manifestations (i.e., observed outcomes or responses) (25–27). It estimates the likelihood of different responses to items by people with different levels of the latent trait being measured (here, the health state as defined by the level answered in each dimension). Then, it helps evaluate how well individual items on assessments work (23). A discriminant coefficient of 1.0 shows that the item discriminates fairly well; persons with low ability (health) have a much smaller chance of having a better score than those with average or high ability. Then, the higher the discrimination coefficient is, the better the capacity of the item to discriminate and the more accurate the information we obtain. A negative discrimination coefficient should be avoided because the probability of having a better score should not decrease with an increase in the respondent's ability (health) (28).

Rasch analysis is a widely used approach in instrument development (7, 29). It is a probabilistic model belonging to the IRT models. The Rasch analysis allows us to evaluate the questionnaire's fit by assessing the person/item separation reliability, the person/item separation index, the standard error measurement (SE), and the infit and outfit statistics. The suggested cutoff values for the person/item separation reliability and the person/item separation index are presented in the corresponding tables (30). Rules of thumb suggest having infit and outfit statistics, which represent the range outside of which the item does not function the way it should function, to be between 0.5 and 1.5 (31). The point-measure correlation for all observations (PTMEASUR-AL), which represents the correlation of the item with the entire group of items, was also calculated. The correlation value (CORR) and the expected value (EXP) predicted by the model are generally positive and close to each other (32). Additionally, the Rasch analysis helped highlight any differential item functioning (DIF) that may occur. DIF methods are usually used to assess the fairness of the items (i.e., if different subgroups of respondents have a different likelihood of answering differently to items) (23, 27, 33). It is generally assessed by calculating the Rasch-Welch, Mantel, and cumulative log-odd in logits (CUMLOR) statistics. According to recommendations on the Winsteps' website, a CUMLOR absolute value of <0.43 would mean that the item presents a negligible difference in functioning; a value between 0.43 and 0.64 would indicate that the item shows a slight to moderate difference in functioning, and a value greater or equal to 0.64 demonstrates the presence of a moderate to large difference in functioning. The items' exclusion/retention was, at this stage, determined according to the psychometric validation results.

Finally, a confirmatory factor analysis (CFA) was conducted by comparing the instrument obtained immediately after the Delphi with the instrument obtained after the validation stage to determine which fit the data better. This was done through a structural equation model (SEM) (34, 35). The root mean square error of approximation (RMSEA), the standardized root mean square residual (SRMR), the comparative fit index (CFI), the Tucker and Lewis Index (TLI), Akaike's information criterion (AIC), and the Bayesian information criterion (BIC) were used. A value of up to 0.08 for the RMSEA was seen as acceptable. However, the closer the value gets to 0, the better it is (3, 36, 37). For the SRMR, values between 0.05 and 0.10 were seen as reflecting an acceptable fit (3, 36, 37). For the CFI and TLI, values of 0.90 or above are considered evidence of acceptable fit (36, 38). Regarding the AIC and BIC, the model with the lower values was considered better (39).

All analyses were performed with Stata BE 17 (Stata Corp, TX, USA) and Winsteps Rasch 5.1.5.2.

### Ethics approval

This study received ethics approval from both the Research Ethics Committees of the CIUSSS de l'Est de l'Île de Montréal (Delphi part) and the CIUSSS de l'Estrie-CHUS (Psychometric part). All participants gave their informed consent before data collection began.

## Results

### Delphi procedure

Between January and April 2021, 18 French-speaking participants took part in the Delphi procedure, and 17 fully completed it, including seven patients, six caregivers, and five citizens (one citizen participated only in round one). The median age was 42 years, and 61% were women. About 50% of the participants claimed to have a good self-rated health state, and 61% suffered from problems that reduced their quality of life (100% in patients and 36% in caregivers and citizens), such as type 1 bipolar disorder, anxiety disorder, autism, schizophrenia, schizoaffective disorder, hypertension, cholesterolemia, fibromyalgia, musculoskeletal disorder, thyroid dysfunction, mild psoriasis, cardiac disorder, diabetes, and cancer. Among caregivers, their relatives suffered from developmental disorders, aging, schizophrenia, anorexia, psychosis, type 1 bipolar disorder, and autism. Table 1 shows the descriptive statistics of the participants. The Delphi procedure was held over four rounds.

**Table 1:** Descriptive statistics of participants.

**Characteristics (%)**	**Participants**
	*n = 18*
Median age at the time of study [range]	42 [19–72]
Women	11 (61.11)
Men	7 (38.89)
Single	8 (44.44)
Married/spouse	8 (44.44)
Separated/divorced	2 (11.11)
***Occupation at the time of study***	
Employed or independent worker	12 (66.67)
Retired	3 (16.67)
Student	2 (11.11)
Unemployed	1 (5.56)
***Education***	
Secondary school or less	1 (5.56)
CEGEP/High school	4 (22.22)
University certificate	2 (11.11)
Bachelor's degree	3 (16.67)
Master's degree	5 (27.78)
Ph.D.	3 (16.67)
***Location zone***	
Urban	15 (83.33)
Rural	3 (16.67)
***General health***	
Have confronted a serious illness at least once	7 (38.89)
Suffer from a physical or mental illness/problem that reduces the quality of life	9 (50)
***Respondents' self-rated health state***	
Excellent	2 (11.11)
Very good	6 (33.33)
Good	9 (50)
Fair	0 (0)
Bad	1 (5.56)

In the first round, 35 items from the literature were presented to the participants. They were first asked to rate each item/dimension on a three-point Likert scale (very important/important/less important). If they answered “less important,” they were requested to provide a reason for the rating. After that, participants were asked if there were any other important dimensions that should be considered in the establishment of the new questionnaire for measuring GHRQoL. Then, they were given 100 points and asked to allocate them to the 35 items (plus additional items they may have suggested) in order of importance. These exercises allowed us to establish a rank from the most important item to the least important. Following these exercises, 28 items/dimensions from the literature were retained, with “Breathing,” “Eating,” “Speech/communication,” “Vision,” “Hearing/listening,” and “Cognitive function” having the highest scores (see Appendix A1 in the Supplementary materials). Ten other items were proposed by the participants (such as “Self-awareness and introspection,” “Access to resources,” and “Trust and respect”). The research team added two more items (“Leisure” and “Maintaining relationships”). The first round ended with 40 proposed items, or dimensions split into 14 meta-dimensions (i.e., groups of related dimensions): “Body functioning,” “Cognition, sense and language,” “Sleep and energy,” “Self-esteem and self-acceptance,” “Pain and physical discomfort,” “Mobility and physical capacity,” “Daily activities and work,” “Social activities and leisure,” “Social and interpersonal relationships,” “Citizenship and social inclusion,” “Autonomy,” “Anxiety and depression,” “Wellbeing,” and “Sexuality and intimacy.” This represented five dimensions for physical health, four dimensions for mental health, four dimensions for social health, and one dimension that could not be classified (i.e., sexuality and intimacy) (6). This round took an average of 22 min for each participant to complete. Table 2 shows the items/dimensions retained in the first round.

**Table 2:** Meta-dimensions and items retained in round 1 of Delphi.

**Proposed items from the literature and ranked by score**	**Participants suggested/added items**	**Meta-dimensions and items retained**	
•Breathing	•Being accepted and understood/being meaningful/being respected	Body functioning	Breathing
•Eating			Eating
• Speech/communication	•Access to resources (money, education, employment, etc.)		Excretion
•Vision		Cognition, sense, and language	Vision
•Hearing/listening			Hearing/listening
•Cognitive function	•Ability to trust/respect		Speech/communication
•Sleep			Cognitive function
•Happiness/wellbeing/satisfaction		Sleep and energy	Sleep
•Vitality/energy	•Spirituality		Vitality/energy
•Excretion		Self-esteem and self-acceptance	Self-confidence/esteem
•Mobility			Introspection and self-knowledge
•Self-confidence/esteem	•Introspection and self-knowledge		Self-acceptance and self-respect
•Interpersonal/social relationships		Pain and physical discomfort	Physical discomfort/pain
•Autonomy/control/dependence		Mobility and physical capacity	Mobility
•Adaptation/coping	•Self-acceptance and self-respect		Dexterity/handling
•Social activities			Self-care
•Enthusiasm/pleasure		Daily activities and work	Usual/daily activities
•Usual/daily activities	•Sense of accomplishment and purpose		Access to resources (money, education, employment, etc.)
•Dexterity/handling		Social activities and leisure	Social activities
•Self-care			Leisure
		Social and interpersonal relationships	Interpersonal/social relationships
•Physical discomfort/pain			Maintaining relationships
•Depression/sadness	•Freedom and self-determination		Being accepted and understood/being meaningful/being respected
•Intimacy/sexuality			Ability to trust/respect
•Anxiety/distress			Loneliness/isolation
•Suicidal ideations	•Psychological suffering and stress	Citizenship and social inclusion	Integration into the community
•Social inclusion/connectedness			Social inclusion/connectedness
•**Terror/fear/panic**			Citizen role
•**Fertility**		Autonomy	Autonomy/control/dependence
Integration into the community	•Citizen role		Adaptation/coping
•**Feeling of being a burden to others**			Freedom and self-determination
Loneliness/isolation		Anxiety and depression	Anxiety/distress
•**Calm/agitation/irritability**	•Leisure**		Depression/sadness
•**Anger**			Suicidal ideations
			Psychological suffering and stress
		Wellbeing	Happiness/wellbeing/life satisfaction
•**Appearance**	•Maintaining relationships**		Enthusiasm/pleasure
			Sense of accomplishment and purpose

Items not retained are in bold.

^**^These items were added by the research team after analysis of participants' comments.

In the second round, levels were specified for each dimension, and we tried to group some items/dimensions together to fit into a single question for each meta-dimension. In this attempt, there were six levels for each meta-dimension. First, participants were asked if they judged the dimensions retained in the first round relevant and if those dimensions showed good equilibrium in the important dimensions in measuring GHRQoL. They all gave a “Yes” response to those two questions. Then, for each meta-dimension and the newly defined levels, participants were asked if they were appropriate and coherent and if the levels' wording was realistic, in addition to clearly showing a decrease in the health state description. Additionally, participants were asked to allocate 100 points to the 14 meta-dimensions in order of importance. It took, on average, 64 min for each participant to complete this phase. For the different meta-dimensions, the agreement level globally fluctuated between 70 and 100% (see Table 3). Many comments came out of this 2nd round and were generally about reformulating and harmonizing the wording in certain meta-dimensions' levels to be more understandable, explicit, and coherent; improving the layout of some level choices that could be confusing when filling out the questionnaire; and splitting certain items/dimensions for more clarity (i.e., grouping various items was not considered to allow for a consistent answer). Regarding the ranking, the meta-dimensions “Body functioning,” “Cognition, sense, and language,” and “Wellbeing” were the most important. “Citizenship and social inclusion” and “Sexuality and intimacy” were ranked as the least important.

**Table 3:** Results after round 2.

**Meta-dimensions as they appear in the questionnaire**	**Formulation appropriateness**	**Response choices coherence**	**Response choices realism**	**Distribution of importance out of 100**
Body functioning	70.6%	70.6%	76.5%	10.26
Cognition, sense, and language	70.6%	70.6%	82.4%	9.32
Sleep and energy	76.5%	70.6%	76.5%	7.50
Self-esteem and self-acceptance	76.5%	70.6%	76.5%	7.03
Pain and physical discomfort	94.1%	94.1%	88.2%	6.28
Mobility and physical capacity	88.2%	94.1%	94.1%	7.56
Daily activities and work	76.5%	88.2%	76.5%	6.90
Social activities and leisure	88.2%	88.2%	88.2%	6.20
Social and interpersonal relationships	82.4%	88.2%	76.5%	6.55
Citizenship and social inclusion	82.4%	91.1%	82.4%	5.72
Autonomy	82.4%	88.2%	100%	7.15
Anxiety and depression	82.4%	100%	100%	6.91
Wellbeing	82.4%	88.2%	88.2%	8.20
Sexuality and intimacy	82.4%	76.5%	88.2%	5.20

Based on participants' comments, we decided to reformulate, split, and replace certain level choices, trying to be as explicit as possible to fit the suggestions. We reorganized the meta-dimensions to allow different response choices for each included dimension in a standard format question when possible. For example, the item/dimension “self-confidence or self-esteem” was separated into two items, “Self-confidence” and “Self-esteem,” and “Deal with unforeseen situations” was transformed into “Adapt to unexpected situations.” Additionally, level wording such as “I never do as many […] activities…” was changed to “I do infinitely fewer activities…” Box 1 shows these changes in the meta-dimension “Body functioning” as an example.

Box 1 **Changes between round two and round three for the dimension “Body functioning”**

The dimension “Body functioning” has undergone the following changes between rounds two and three. In the 2nd round, levels have been proposed for the items retained in round one. For this dimension, the proposed levels were as follows:

I have no difficulty breathing, eating, or urinating/defecating.

I have some difficulty breathing, eating, or urinating/defecating.

I have difficulty breathing, eating, or urinating/defecating.

I have great difficulty breathing, eating, or urinating/defecating.

I have very great difficulty breathing, eating, or urinating/defecating.

I am in physical distress and/or need assistance to urinate/defecate.

In the 3rd round, a new answering format was proposed with some changes in the levels' wording as well as in the wording of some items.

**Table:** 

	Breathing	Eating	Evacuating (urine and fecal matter)
I have no difficulty with… I have very little difficulty with… I have little difficulty with… I have a lot of difficulty with…. I have tremendous difficulty with… I need assistance with…

The third round allowed the research team to propose a new format and wording for levels according to suggestions received in the previous round. Participants were asked a single question for each meta-dimension to establish if the new response choices offered were relevant and coherent and if they correctly reflected the topic of the corresponding meta-dimension. They were also asked to formulate suggestions to improve the text if necessary. Participants agreed between 82 and 100% with the following question: “Do you find that the response options offered reflect the chosen theme [i.e., meta-dimension] and are relevant and consistent in their formulation?” (see Table 4). The degree of agreement was higher than in the previous round, indicating that the questionnaire's format change was necessary. The mean time of completion in this round was 19 min.

**Table 4:** Agreement variation between round 2 and round 3.

**Meta-dimensions**	**Round 2 agreement level (mean)**	**Round 3 agreement level**	**Variation**
Body functioning	73%	94%	+30%
Cognition, sense, and language	75%	88%	+18%
Sleep and energy	75%	82%	+11%
Self-esteem and self-acceptance	76%	82%	+8%
Pain and physical discomfort	92%	94%	+2%
Mobility and physical capacity	92%	94%	+2%
Daily activities and work	80%	88%	+10%
Social activities and leisure	86%	94%	+9%
Social and interpersonal relationships	82%	82%	0%
Citizenship and social inclusion	86%	100%	+16%
Autonomy	90%	94%	+4%
Anxiety and depression	94%	100%	+6%
Wellbeing	86%	100%	+16%
Sexuality and intimacy	82%	82%	0%
**All dimensions**	**84%**	**91%**	**+9%**

Percentages in the first column are the average agreement level of respondents for the three questions related to the appropriateness, consistency, and credibility of the retained meta-dimensions and levels' wording.

A shift in the general agreement level compared to the previous round (i.e., between 70 and 100% for the three questions) can be imputed to the format's change that allowed an improvement in the understanding and the coherence of the response' choices, was observed (see Table 4). This allowed an average shift of 9% in the agreement level (i.e., from 84% in round two to 91% in round three). Nevertheless, some comments were recorded and were mostly about spelling correction, wording (reformulation of some items with better fitting terms/expressions, the addition of some examples to make the item's title more explicit), and modification of a few response choices.

Based on these comments, some adjustments were made. The meta-dimension “Insecurity and fear” was added to the questionnaire with two items and six levels each, as suggested by participants. The descriptor “Physical” was added to the initial item “Pain and discomfort” (i.e., now “Physical pain and discomfort”). An intermediary choice level “I have difficulty with…” was added to the “Mobility and physical disability,” “Cognition, sense, and language,” “Body functioning,” “Self-esteem and self-acceptance,” and “Daily activities and work” items. Additionally, the level “I have problems with…” was added to the “Sleep and energy” and “Self-esteem and self-acceptance” items. For the meta-dimension “Sexuality and intimacy,” the level “I am very satisfied of…” was also added. The last choice level for “Self-esteem and self-acceptance” was modified to “I feel like a loser and never fit in. I have no…” and “I do not deserve any consideration. I am completely incapable of…”

In the fourth round of the Delphi procedure, a revised version of the questionnaire was proposed based on the comments from the previous round (see Appendix A2 in the Supplementary materials for an extensive list of changes made between rounds two and four). In this last round, participants gave their opinion related to the question, “In general, in relation to the objective of the study, which is to develop an instrument to measure global health-related quality of life (GHRQoL), do you think that the choice of dimensions and response options offered are relevant and appropriate?” All of them agreed by answering “Yes.” They were also asked to give their level of agreement with the statement: “The dimensions and response options offered are relevant and appropriate.” Participants agreed by 95% on average (agreement levels ranged between 85 and 100%). The comments showed overall satisfaction and agreement with participants on the last version of the questionnaire proposed. A consensus was then attained, and no modifications were needed after round four. The final questionnaire derived through the Delphi procedure included 15 dimensions and 36 items with five to seven levels each.

### Psychometric validation

From April to June 2021, an online survey with the general Quebecker population was held to proceed to the psychometric validation of the dimensions retained in the Delphi procedure. For this survey, 3,028 French-speaking participants were recruited to complete the questionnaire, which comprised 15 dimensions and 36 items of five to seven levels each (See the right part of the table in Appendix A2 in the Supplementary materials). Through the screening procedure, 755 observations were dropped because they did not satisfy the data requirements (i.e., incomplete). The validation stage involved the complete remaining 2,273 observations. Table 5 shows the descriptive statistics of the respondents considered in the psychometric validation stage.

**Table 5:** Descriptive statistics of respondents.

**Characteristics (%)**	**Participants**
	*n = 2,273*
Median age (year) [range]	47 [17–94]
BMI (kg/m2) [range]	26.79 [8.19–97.42]
***Gender***	
Women	1,107 (48.71)
Men	1,160 (51.03)
Intersex/other	6 (0.26)
***Status***	
Single	784 (34.49)
Married/spouse	1,225 (53.89)
Separated/divorced	179 (7.88)
Widower	85 (3.74)
***Occupation***	
At home	117 (5.15)
Employed or independent worker	1,117 (49.14)
Retired	701 (30.84)
Sick leave/maternity	54 (2.38%)
Student	162 (7.13)
Unemployed	119 (5.24)
Other	3 (0.13)
***Education (general information)***	
Pursued study after minimum age (16 years old)	1,588 (69.86)
Got a diploma/certification	1,840 (80.95)
***Education (highest level)***	
Primary school diploma	34 (1.50)
Secondary	514 (22.61)
Diploma of professional study	232 (10.21)
High school	611 (26.88)
University certificate	215 (9.46)
Bachelor's degree	411 (18.08)
Master's degree	183 (8.05)
Ph.D.	70 (3.08)
Others	3 (0.13)
***Household income***	
<9,999$	91 (4.00)
10,000–34,999$	532 (23.41)
35,000–44,999$	226 (9.94)
45,000–54,999$	227 (9.99)
55,000–64,999$	160 (7.04)
65,000–74,999$	163 (7.17)
75,000–84,999$	172 (7.57)
85,000–99,999$	194 (8.53)
100,000–119,999$	220 (9.68)
120,000–149,999$	166 (7.30)
> 150,000$	122 (5.37)
***Habitation***	
Urban	1,623 (71.40)
Rural	650 (28.60)
Homeowner	1,421 (62.52)
Tenant	852 (37.48)
***Country of birth***	
Canada	2,013 (88.56)
Other	260 (11.44)
***Health problem***	
Have been at least once confronted with a serious illness	616 (27.10)
Self-reported problems affecting HRQoL	623 (27.41)
***Self-rated health state***	
Excellent	340 (14.96)
Very good	863 (37.97)
Good	832 (36.60)
Fair	191 (8.40)
Bad	47 (2.07)

The data analysis displayed a Cronbach's alpha of 0.94, which is in the targeted range (0.75–0.95). No items appeared to increase the Cronbach's alpha by 2% when removed (see Table 6). Regarding item-test correlation, all items showed good coefficients between 0.36 and 0.74, except “Safety,” which had a low coefficient of 0.21. “Safety” was also the only item to register a correlation coefficient <0.2 for the item-rest correlation. No items were seen to have inter-item coefficients <0.2 or >0.4.

**Table 6:** Cronbach's alpha and item correlations.

**Items**	**Item-test correlation**	**Item-rest correlation**	**Inter-item correlation**	**Cronbach's alpha if item removed**
Breathing	0.4762	0.437	0.3042	0.9387	
Eating	0.5204	0.4832	0.3027	0.9383	
Evacuating	0.5135	0.476	0.303	0.9383	
Cognition	0.603	0.5704	0.2999	0.9375	
Senses	0.5618	0.5268	0.3013	0.9379	
Language	0.5659	0.5312	0.3012	0.9378	
Sleep	0.5132	0.4757	0.303	0.9383	
Energy	0.5874	0.5538	0.3004	0.9376	
Confidence	0.606	0.5736	0.2998	0.9374	
Accepting myself	0.6217	0.5902	0.2993	0.9373	
Pain	0.5356	0.4992	0.3022	0.9381	
Discomfort	0.6036	0.571	0.2999	0.9375	
Performing strenuous activities	0.4919	0.4534	0.3037	0.9385	
Performing moderate activities	0.6042	0.5717	0.2999	0.9375	
Selfcare	0.5883	0.5548	0.3004	0.9376	
Daily activities	0.6588	0.6297	0.298	0.9369	
Work or study	0.68	0.6523	0.2973	0.9367	
Social	0.4983	0.4601	0.3035	0.9385	
Leisure	0.5919	0.5586	0.3003	0.9376	
Accepted and listened	0.6542	0.6248	0.2981	0.937	
Affection and support	0.6189	0.5873	0.2993	0.9373	
Engaged in my role	0.4539	0.4136	0.305	0.9389	
Integrated into society	0.4898	0.4511	0.3038	0.9385	
Autonomous	0.3626	0.3189	0.3081	0.9397	
Face unexpected situations	0.6019	0.5691	0.2999	0.9375	
Sad or depressed	0.651	0.6213	0.2982	0.937	
Anxious or stressed	0.674	0.6458	0.2975	0.9368	
Angry or irritated	0.5988	0.566	0.3	0.9375	
Safety	0.2104	0.1628	0.3133	0.9411	
Scared or worried	0.5787	0.5447	0.3007	0.9377	
Fulfilled	0.638	0.6075	0.2987	0.9371	
Useful	0.7218	0.697	0.2958	0.9363	
Satisfied	0.7358	0.712	0.2953	0.9362	
Sex life	0.4775	0.4383	0.3042	0.9387	
Intimacy	0.535	0.4986	0.3022	0.9381	
Sexual identity	0.5584	0.5232	0.3014	0.9379	
Test scale			0.3012	0.9394	

Floor and ceiling effects were also explored, and approximately 1.28% of the participants chose the best possible answer in all response choices (i.e., first choice or best health level). No respondent always chose the worst possible answer (i.e., worst health level). Regarding the results far below the cutoff point of 15% stated earlier, we can conclude that our questionnaire does not suffer from floor or ceiling effects.

The pattern described by the correlation analysis was intended to be verified by PCA. Prior to that, the Bartlett test of sphericity and the KMO test were performed. The Bartlett test of sphericity showed a chi-square value equal to 40671.28 (*p*-value = 0.000), and the KMO test measured the sampling adequacy and was equal to 0.95. The significance of the former and the fact that the latter was >0.5 were some indications of the presence of sufficient intercorrelations in our data to conduct the factor analysis.

An unrotated PCA was performed to identify components retained in the varimax rotation. Six components recorded eigenvalues >1 and explained 58.12% of the total variance. The PCA with varimax rotation determined the contribution of each item to the explained variance of each component. Of the 36 items, 34 showed a coefficient >0.2. Additionally, it was observed that most items belonging to the same meta-dimension were located in the same component (see Table 7). In this way, component one gathered the items of the meta-dimensions “Self-esteem and self-acceptance” and “Depression, anxiety, and anger.” Component two clustered the meta-dimensions “Body functioning” and “Cognition, senses, and language,” and components three, five, and six regrouped the items of the meta-dimensions “Sleep and energy,” “Social and leisure activities” and “Citizenship and social inclusion,” respectively. Nevertheless, some other meta-dimensions had their items dispersed among different components. This was the case for the meta-dimensions “Mobility and physical disability,” “Social and interpersonal relationships,” “Autonomy and adaptation,” and “Sexuality and intimacy.” It was interesting to note that the item “Bathe, dress, or feed myself” was located in the same component as that contained the meta-dimensions “Body functioning” and “Cognition, senses, and language.” This shows that an aspect generally considered very specific in conventional questionnaires (e.g., EQ-5D) can be considered in the measurement of physical health, such as what is done in the SF-6Dv2. The two items with coefficients <0.2 were “Safety” and “Scared, or worried,” which form the meta-dimension “Insecurity and fear.” These items are also located in separate components (2nd and 6th components).

**Table 7:** Items ponderation in PCA with varimax rotation.

**Meta-dimensions**	**Variables**	**Comp1**	**Comp2**	**Comp3**	**Comp4**	**Comp5**	**Comp6**
Body functioning	Breathing	−0.04	**0.30**	0.00	−0.05	0.08	−0.01
	Eating	0.02	**0.36**	−0.07	−0.04	−0.02	−0.04
	Evacuating	−0.02	**0.34**	−0.01	−0.02	−0.04	0.00
Cognition, senses, and language	Cognition	0.06	**0.20**	0.07	0.01	0.08	−0.07
	Senses	−0.04	**0.27**	0.09	0.05	0.00	−0.01
	Language	0.03	**0.36**	−0.08	−0.02	−0.03	0.00
Sleep and energy	Sleep	0.17	−0.07	**0.31**	−0.08	−0.03	−0.06
	Energy	0.17	−0.08	**0.31**	−0.03	0.01	−0.03
Self–esteem and self–acceptance	Confidence	**0.35**	−0.03	0.04	−0.06	−0.12	0.02
	Accepting myself	**0.32**	−0.01	0.06	−0.02	−0.11	−0.01
Pain and physical discomfort	Pain	−0.01	−0.03	**0.50**	0.02	−0.07	0.00
	Discomfort	0.02	−0.01	**0.48**	0.02	−0.09	0.01
Mobility and physical disability	Performing strenuous activities	−0.15	0.05	**0.36**	0.05	0.19	0.05
	Performing moderate activities	−0.12	0.21	**0.28**	0.03	0.09	0.03
	Selfcare	−0.01	**0.36**	−0.01	−0.02	−0.01	0.01
Daily activities and work	Daily activities	−0.02	**0.22**	0.18	−0.01	0.13	−0.03
	Work or study	0.04	**0.24**	0.14	−0.01	0.04	−0.04
Social and leisure activities	Social	0.03	−0.01	−0.05	−0.04	**0.60**	0.06
	Leisure	0.02	0.04	−0.01	−0.03	**0.52**	0.08
Social and interpersonal relationships	Accepted and listened	**0.21**	0.01	0.00	0.04	−0.02	0.19
	Affection and support	0.15	0.01	−0.02	**0.22**	−0.06	0.19
Citizenship and social inclusion	Engaged in my role	−0.05	−0.04	0.03	−0.02	0.12	**0.59**
	Integrated into society	0.00	0.01	−0.02	−0.03	0.02	**0.59**
Autonomy and adaptation	Autonomous	0.08	0.06	0.00	−0.06	−0.21	**0.34**
	Face unexpected situations	**0.22**	0.03	0.07	−0.04	−0.10	0.13
Depression, anxiety, and anger	Sad or depressed	**0.33**	−0.04	0.02	−0.02	0.12	−0.10
	Anxious or stressed	**0.35**	0.02	−0.03	−0.06	0.11	−0.12
	Angry or irritated	**0.26**	0.08	−0.07	−0.01	0.06	−0.06
Insecurity and fear	Safety	0.11	**0.17**	−0.05	−0.12	−0.32	0.11
	Scared or worried	−0.27	−0.07	0.06	0.05	−0.11	**0.12**
Wellbeing	Fulfilled	**0.23**	−0.07	−0.01	0.17	0.12	0.04
	Useful	**0.20**	0.03	−0.04	0.15	0.10	0.08
	Satisfied	**0.24**	0.04	−0.04	0.13	0.06	0.05
Sexuality and intimacy	Sex life	−0.02	−0.04	0.03	**0.62**	0.00	−0.03
	Intimacy	0.00	0.02	−0.01	**0.62**	−0.04	−0.02
	Sexual identity	0.03	**0.27**	−0.12	0.23	−0.07	0.01
Explained variance value	Proportion	0.17	0.15	0.09	0.06	0.06	0.05
	Cumulative	0.17	0.32	0.41	0.47	0.53	0.58

Highest item coefficients are in bold.

The model fit was checked using the IRT model with graded responses and Rasch analysis. The discriminant capacity of items was assessed, and all the discriminant coefficients appeared to be high (>1) and significant, except for the items “Safety” and “Scared or worried,” which presented low and negative discrimination coefficients, respectively.

The same pattern was observed with Rasch analysis. The SE was very low, with a value of 0.2, and the item separation reliability (1.00), item separation index (15.80), person separation reliability (0.83), and person separation index (2.18) were all satisfactory (see Table 8). The items “Safety” and “Scared, or worried” appeared to register high infit and outfit statistics values (see Table 9). These items also had very different CORR and EXP-values. In addition, “Scared or worried” registered a negative CORR. The item “Autonomous” also showed infit and outfit values out of range but, contrary to the previous items, had positive and close CORR and EXP coefficients that indicated no major trouble with the item. Regarding the DIF, two items (“Evacuating” and “Accepting myself”) were apparently associated with moderate to large DIF. No alarming values (far <0.64) were recorded (see Table 10).

**Table 8:** Standard error of measurement, item/person separation index and item/person separation reliability.

**Psychometric testing**	**Value**	**Suggested cutoff**
Standard error of measurement (mean)	0.20	The smaller, the better
Item separation reliability from Rasch	1.00	>0.7
Item separation index from Rasch	15.80	>2
Person separation reliability from Rasch	0.83	>0.7
Person separation index from Rasch	2.18	>2

**Table 9:** Rasch analysis results.

	**INFIT**		**OUTFIT**		**PTMEASUR–AL**		**Discrimination**
	**MNSQ**	**ZSTD**	**MNSQ**	**ZSTD**	**CORR**.	**EXP**.	
Breathing	1.79	9.9	1.28	4.3	0.38	0.33	1.43
Eating	1.9	9.9	1.31	4.94	0.41	0.35	1.67
Evacuating	1.65	9.9	1.37	6.16	0.42	0.39	1.51
Cognition	1.15	2.85	0.8	−3.75	0.48	0.35	2.12
Senses	1.08	1.82	0.94	−1.12	0.47	0.4	1.50
Language	1.33	5.53	0.93	−1.17	0.45	0.34	1.89
Sleep	0.97	−0.83	1.07	1.46	0.48	0.49	1.30
Energy	0.8	−6.9	4.72	−7.25	0.56	0.51	1.74
Confidence	1.23	6.25	1	0.06	0.52	0.48	1.99
Accepting myself	1.17	4.5	0.94	−1.22	0.53	0.46	2.13
Pain	0.87	−4.3	1.83	−4.13	0.51	0.5	1.32
Discomfort	0.74	−8.1	3.67	−7.91	0.54	0.46	1.66
Performing strenuous activities	1.31	9.33	1.21	4.72	0.48	0.53	1.16
Performing moderate activities	1.3	7.33	0.99	−0.25	0.52	0.45	1.78
Self–care	1.7	9.9	1.04	0.74	0.46	0.36	2.23
Daily activities	1.36	7.26	0.85	−2.94	0.52	0.39	2.64
Work or study	1.33	7.29	0.88	−2.4	0.54	0.42	2.55
Social	0.96	−1.3	11.14	3.37	0.49	0.54	1.18
Leisure	0.76	−8.7	0.87	−3.18	0.57	0.54	1.47
Accepted and listened	0.45	−9.9	0.46	−9.9	0.57	0.4	2.10
Affection and support	0.44	−9.9	0.47	−9.9	0.57	0.43	1.83
Engaged in my role	1.26	7.9	1.27	6.11	0.48	0.53	1.21
Integrated into society	1.2	6.12	1.21	4.62	0.49	0.52	1.36
Autonomous	**1.94**	9.9	**2.04**	9.9	0.36	0.45	1.29
Face unexpected situations	1.05	1.36	0.85	−3.17	0.52	0.45	2.05
Sad or depressed	0.4	−9.9	0.45	−9.9	0.57	0.45	1.99
Anxious or stressed	0.42	−9.9	0.49	−9.9	0.59	0.47	2.03
Angry or irritated	0.45	−9.9	0.48	−9.9	0.55	0.47	1.71
Safety	**1.69**	9.9	**3.19**	9.9	**0.2**	**0.57**	**0.38**
Scared or worried	**2.32**	9.9	**4.07**	9.9	**−0.39**	**0.65**	**−1.68**
Fulfilled	0.63	−9.9	0.66	−8.85	0.6	0.5	2.09
Useful	0.52	−9.9	0.49	−9.9	0.65	0.49	2.53
Satisfied	0.53	−9.9	0.49	−9.9	0.67	0.52	2.65
Sex life	1.25	8.28	1.16	4.15	0.51	0.58	1.20
Intimacy	1.22	7.29	1.12	3.1	0.54	0.57	1.42
Sexual identity	1.36	7.89	1.07	1.27	0.46	0.41	1.49
MEAN	1.13	1.4	1.08	−1.2			
P.SD	0.48	7.9	0.71	6.2			

Values out of range in bold.

**Table 10:** Fit statistics and item selection.

		**Rasch–Welch**		**Mantel**	**Siz**		**Item exclusion or retention**
**Meta–dimensions**	**Items**	**t**	**Prob**	**Chi–square**	**Prob**.	**CUMLOR**	
Body functioning	Breathing	−4.88	0.00	17.14	0.00	−0.56	✓
	Eating	−5.47	0.00	13.98	0.00	−0.53	✓
	Evacuating	−7.06	0.00	29.23	0.00	−0.67	✓
Cognition, senses, and language	Cognition	0.00	1.00	1.24	0.27	−0.14	✓
	Senses	−4.11	0.00	15.69	0.00	−0.43	✓
	Language	−5.17	0.00	23.50	0.00	−0.63	✓
Sleep and energy	Sleep	5.42	0.00	24.84	0.00	0.46	✓
	Energy	3.68	0.00	13.12	0.00	0.34	✓
Self–esteem and self–acceptance	Confidence	5.03	0.00	17.43	0.00	0.43	✓
	Accepting myself	7.60	0.00	43.77	0.00	0.72	✓
Pain and physical discomfort	Pain(s)	3.29	0.00	12.67	0.00	0.33	✓
	Discomfort	2.94	0.00	8.36	0.00	0.28	✓
Mobility and physical disability	Performing strenuous activities	3.03	0.00	5.24	0.02	0.21	✓
	Performing moderate activities	0.00	1.00	0.02	0.88	−0.02	✓
	Selfcare	−4.87	0.00	10.89	0.00	−0.47	✓
Daily activities and work	Daily activities	0.00	1.00	0.73	0.39	0.11	✓
	Work or study	−1.69	0.09	0.92	0.34	−0.12	✓
Social and leisure activities	Social	4.18	0.00	17.01	0.00	0.35	✓
	Leisure	0.00	1.00	0.83	0.36	0.08	✓
Social and interpersonal relationships	Accepted and listened	1.54	0.12	4.60	0.03	0.21	✓
	Affection and support	−1.46	0.14	6.19	0.01	−0.24	✓
Citizenship and social inclusion	Engaged in my role	−2.35	0.02	6.90	0.01	−0.24	✓
	Integrated into society	0.00	1.00	1.47	0.22	−0.11	✓
Autonomy and adaptation	Autonomous	−4.02	0.00	10.95	0.00	−0.36	✓
	Face unexpected situations	0.00	1.00	0.05	0.83	0.02	×
Depression, anxiety, and anger	Sad or depressed	4.43	0.00	53.58	0.00	0.69	✓
	Anxious or stressed	4.01	0.00	40.48	0.00	0.57	✓
	Angry or irritated	0.00	1.00	6.40	0.01	0.24	✓
Insecurity and fear	Safety	−5.32	0.00	9.52	0.00	−0.26	×
	Scared or worried	−7.38	0.00	20.68	0.00	−0.41	×
Wellbeing	Fulfilled	2.73	0.01	6.21	0.01	0.22	✓
	Useful	1.68	0.09	2.89	0.09	0.15	✓
	Satisfied	1.44	0.15	1.58	0.21	0.11	✓
Sexuality and intimacy	Sex life	−1.76	0.08	3.04	0.08	−0.15	✓
	Intimacy	0.00	1.00	1.99	0.16	−0.12	✓
	Sexual identity	−3.65	0.00	11.69	0.00	−0.38	✓

These results confirm our previous findings. Our questionnaire displayed many satisfying results that confirmed its goodness of fit. Nevertheless, some items (*n* = 3) were deleted to end up with the best possible items that would form a questionnaire able to measure the GHRQoL of respondents in the best way (see Table 10). These items showed some major inaccuracies through the validation process, such as “Safety” and “Scared or worried” (forming the meta-dimension “Insecurity and fear”). The meta-dimension “Autonomy and adaptation” revealed some dysfunction where in the PCA, the items “Autonomous” and “Face unexpected situations” were in separate components, with “Autonomous” contributing more to the component's variance with items of the meta-dimension “Citizenship and social inclusion.” This suggested the deletion of the item “Face unexpected situations” and the reorganization of the meta-dimension “Citizenship and social inclusion” with the addition of the item “Autonomous.”

A CFA was finally performed to confirm the best model (instrument). To do so, the instrument obtained after the Delphi (Model 1) was compared to the reorganized instrument (i.e., obtained after the validation stage – Model 2). The results can be seen in Table 11. It was generally observed that for every criterion considered regarding the different cutoff points, the model obtained after the validation stage showed a better fit to the data. This confirms our previous results that led to the deletion/reorganization of some items/meta-dimensions (see Appendix A3 in the Supplementary materials for the figure of the CFA Model 1 structure and results). This final version also displayed high internal reliability with an unchanged Cronbach's alpha value of 0.94.

**Table 11:** Statistics resulting from the CFA.

	**Models**	
	Model 1*	Model 2**
RMSEA	0.065	0.059
SRMR	0.135	0.068
CFI	0.882	0.914
TLI	0.851	0.891
AIC	259384.065	235072.414
BIC	260541.294	236086.421
N	2,273	2,273

^*^Model 1 is the estimated model using the structure of the instrument obtained after the Delphi procedure (i.e., an instrument with 15 dimensions and 36 items with five to seven levels each).

^**^Model 2 is the estimated model using the structure of the instrument obtained after the validation stage (i.e., an instrument with 13 dimensions and 33 items with five to seven levels each).

The questionnaire ended with 13 meta-dimensions and 33 items with five to seven levels each. The questionnaire allows the measurement of GHRQoL in a balanced way by providing five meta-dimensions for physical health, four meta-dimensions for mental health, three meta-dimensions for social health and one meta-dimension for sexuality and intimacy (see Appendix A4 in the Supplementary materials for a translation of the 13-MD in English and Appendix A5 for the original instrument in French).

## Discussion

This study describes the steps and results of the development of the 13-MD, a generic instrument that comprises 33 items with five to seven levels each to measure GHRQoL. This new instrument was developed to compensate for the imbalance noted in the composition of existing generic instruments (7). The 13-MD offers various items forming meta-dimensions covering the main important domains of health. It includes five meta-dimensions for physical health, four meta-dimensions for mental health, three meta-dimensions for social health, and one meta-dimension for sexuality and intimacy.

A Delphi procedure allowed us to gather representatives of various health stakeholders and to collect their views and expectations regarding what needs to be a balanced and complete instrument. Four rounds were necessary to establish a core structure that comprises 15 meta-dimensions with 36 items that would be amenable to further psychometric tests.

The PCA, IRT, Rasch analysis, and other psychometric tests conducted in this study confirmed the good structure obtained from the Delphi procedure, which reinforces our confidence in the instrument's ability to measure what it intends. The data obtained from the Quebecker general population allowed us to see the consistency of our questionnaire and to detect potentially misfunctioning items. At the end of the process, some items (i.e., three items) were excluded to maximize the questionnaire fit and offer users the best version of the instrument for GHRQoL measurement. Specifically, the items “Safety,” “Scared or worried” (forming the meta-dimension “Insecurity and fear”), and “Face unexpected situations” were dropped.

Neither minimizing the existing good quality instruments nor denying the importance of physical wellbeing in a person's life, the 13-MD aims to represent as a whole and in a balanced way the meta-dimensions that reflect significant life domains. Every aspect that could impact people's GHRQoL was considered and measured. The balance between meta-dimensions was a key point in the construction of the 13-MD, and no absolute predominance of one meta-dimension was sought. We believe health to be complex and every aspect of life to be important (10). That is why making available an instrument that could measure HRQoL in all its forms regardless of people's cultural backgrounds was a priority. The 13-MD is thus meant to be usable by anyone.

Compared to existing instruments, the 13-MD distinguishes itself by its structure. A large number of response choices (i.e., five to seven levels for each item) permits 1.42E+26 (i.e., 5^5^x6^15^x7^13^) possibilities. This is more than what is allowed by the Assessment Quality of Life-8 (AQoL-8D) with its 3.56E+24 possibilities (7, 40). A large number of possible combinations allows for the consideration of several possible and complex health states. It could lead to a more precise measure and better discrimination between respondents, as shown in the results. The AQoL-8D is a generic instrument with approximately the same number of items as the 13-MD (i.e., 35 and 33 items, respectively), but the latter differs clearly in its structure and composition. In fact, the AQoL-8D sought to emphasize the psychosocial aspect of health with five meta-dimensions compared to three meta-dimensions for the physical aspect (41). With eight meta-dimensions for 35 items in the AQoL-8D, we can also observe some meta-dimensions containing items that could be seen as transversal, that is, not exclusive to the meta-dimension(s) in which they are embedded (e.g., the items “intimacy” and “pleasure” with the meta-dimensions “Relationships,” and “Happiness,” respectively). In that way, for example, someone not happy with his or her intimacy due to physical disturbances could be seen as only having relationship issues. In contrast, with fewer items, the 13-MD aimed to consider all aspects of health globally without overrepresentation. Additionally, the 13-MD provides a greater diversity of meta-dimensions to be as specific and precise as possible. Each meta-dimension only contains items concerned with the topic addressed in the meta-dimension. In fact, the “Sexuality and intimacy” meta-dimension was sorted as an independent meta-dimension and contained items exclusive to it (6). With this structure, score variation within individuals or for the same individual within periods can be specifically seen and explained by locating the meta-dimension(s) behind these variations because they contain items approaching the same topic. In addition, the 13-MD covers unconsidered points in the AQoL-8D but no less important for HRQoL measurement, such as citizenship, affection and support, and self-acceptance.

The systematic review by Touré et al. (7) helps to consider the 13-MD as the only generic instrument to present more than five levels by the item on an instrument with more than 10 meta-dimensions. This systematic review recorded, among the wider instruments that cover most dimensions, that only the 15-dimension (15D) (42) and the Quality of WellBeing Self-Administered (QWB-SA) (43) have dimensions related to sexual activity; however, the 15D has no dimension relative to the social domain, and the QWB-SA does not measure social inclusion/connectedness. Additionally, the 13-MD appears to be the instrument that adds new dimensions to the social domain while incorporating conventional dimensions found in other instruments. Indeed, the question of citizenship and role in society is, to the best of our knowledge, not considered in other generic instruments measuring GHRQoL and, subsequently, QALY. This point addressed by this new dimension has been recognized as a burning issue with the recent COVID-19 pandemic and lockdown sanitary measures. As the primary purpose of the 13-MD was to offer a balanced instrument able to measure GHRQoL, it also emphasizes the mental aspect of health compared to commonly used generic instruments. For example, the EQ-5D-5L and the SF-6Dv2 have fewer than one in five dimensions related to mental health, whereas the 13-MD contains four meta-dimensions dedicated to it (7).

On the other hand, the 13-MD differs from other instruments in its layout. We sought a format to ensure the questionnaire was easily read and completed. The table format proposed helps in easily navigating through the questionnaire and seeing the existing response options for each item. Thus, fewer filling errors are expected to result in more quality responses and higher survey filling levels.

### Limits

Considering the large variety of existing psychometric tests, we acknowledge that further tests could be performed, such as a test-retest (44). Additionally, it would be interesting to validate the 13-MD on larger samples from different parts of the world. We could then better challenge the instrument's performance and reliability by seeing if it performs differently. Doing so will make it possible to assess the instrument's cross-cultural validity (45). However, these suggestions need time and resources we do not currently have. For the moment, we ensured that the 13-MD went through a strict and rigorous development process. The results obtained in the psychometric tests confirm this fact and show that the 13-MD is consistent and reliable. However, we acknowledge that, like other well-known instruments, the 13-MD may need further improvements (3, 46). Its future use by researchers and decision-makers will thus give us more information on its functionality and allow us to make some adjustments if needed. Additionally, one concern may be related to the fact that the version proposed in this study was originally in French, and the English version in Appendix A4 in the Supplementary materials has not undergone back translations (47, 48). This process would ensure that the English version correctly reflects the meaning of the original French version, obtained through the Delphi process and validated with the general population of Quebec.

Due to the initial preoccupation with measuring the GHRQoL as fully as possible, the 13-MD is a lengthy instrument (33 items with five to seven levels each). It can be time-consuming for respondents to fill out compared to some other well-known generic instruments (i.e., SF-6Dv2, EQ-5D-5L). This can make respondents or researchers reticent to complete or use the new instrument, although it will be at the expense of less sensitivity. We acknowledge that this can be a real obstacle to its expansion and appropriation by the potential research community, which is why a shorter version may be developed later.

### Next steps

Acknowledging the specificities relative to each language, it would be useful to conduct a back translation of the English version of the 13-MD presented in this study. This step could involve native English and French speakers fluent in both languages. The aim would be to determine the best quality language for the 13-MD so that it could be well understood and filled out by the concerned population.

To be usable in the QALY calculation, the 13-MD must also pass the valuation stage of developing a preference-based conversion algorithm using some elicitation methods (49, 50). This will allow calculating a score with the 13-MD. Once this is done, it would be interesting to compare the value set defined by the 13-MD with other well-known generic instruments (e.g., EQ-5D, SF-6Dv2, HUI-3). Testing and comparing those differences, if any exist, will allow us to establish how the incorporation of items that were not well considered in early instruments affects HRQoL with score variation between instruments. It will also test the convergent validity of the 13-MD, which was not done in this study, considering that its value set is not yet developed.

## Conclusion

The existing imbalance among generic instruments regarding their composition, where dimensions related to physical aspects of health are depicted more often, and the fact that few instruments consider dimensions relative to the mental and social domains were sufficient motivations to develop a more balanced instrument to measure GHRQoL. A Delphi procedure was held in four stages to establish a consistent structure that passed a series of psychometric validation tests and showed satisfying results. The 13-MD, with 33 items or dimensions with five to seven levels each, is a balanced questionnaire considering important aspects of GHRQoL. The 13-MD comprises five meta-dimensions for physical health, four meta-dimensions for mental health, three meta-dimensions for social health, and one meta-dimension for sexuality and intimacy. The upcoming valuation stage will allow the 13-MD to be fully operational for use in the QALY calculation.

## Data availability statement

The original contributions presented in the study are included in the article/Supplementary material, further inquiries can be directed to the corresponding author.

## Ethics statement

This study received ethics approval from both the Research Ethics Committees of the CIUSSS de l'Est de lÎle de Montréal (Delphi part) and the CIUSSS de l'Estrie-CHUS (Psychometric part). All participants gave their informed consent before data collection began.

## Author contributions

TGP and AL conceived the study. TGP, AL, and MT conducted the survey. TGP and MT analyzed the data and wrote the manuscript. All authors contributed to the article and approved the submitted version.

## Funding

This study was funded by the Unité Soutien SSA Québec and the Fondation de l'IUSMM and also supported from the CIRANO.

## Conflict of interest

The authors declare that the research was conducted in the absence of any commercial or financial relationships that could be construed as a potential conflict of interest.

## Publisher's note

All claims expressed in this article are solely those of the authors and do not necessarily represent those of their affiliated organizations, or those of the publisher, the editors and the reviewers. Any product that may be evaluated in this article, or claim that may be made by its manufacturer, is not guaranteed or endorsed by the publisher.
