# Supplementary material for: Development of a balanced instrument to measure global health-related quality of life: The 13-MD
Source: Front Psychiatry. 2022 Sep 6;13:837510. doi: 10.3389/fpsyt.2022.837510 (PMC9485584; doi:10.3389/fpsyt.2022.837510)
Supplement: Supplementary file 1 [file Data_Sheet_1.docx]

**Appendix**

**A1**: Ranking results after the Delphi round 1

| Dimension | Points out of 100 | Very important | Important | Less important |
| --- | --- | --- | --- | --- |
| Breathing | 4,75 | 77,78 | 22,22 | 0 |
| Eating | 4,49 | 61,11 | 38,89 | 0 |
| Speech/Communication | 4,30 | 83,33 | 16,67 | 0 |
| Vision | 4,26 | 83,33 | 16,67 | 0 |
| Hearing/Listening | 4,06 | 83,33 | 16,67 | 0 |
| Cognitive function | 4,04 | 89,89 | 11,11 | 0 |
| Sleep | 3,90 | 72,22 | 27,78 | 0 |
| Well-being/Happiness/Satisfaction | 3,87 | 77,78 | 22,22 | 0 |
| Vitality/Energy | 3,85 | 66,67 | 33,33 | 0 |
| Excretion | 3,35 | 66,67 | 27,78 | 5,56 |
| Mobility | 3,35 | 72,22 | 27,78 | 0 |
| Self-confidence/Esteem | 3,20 | 66,67 | 33,33 | 0 |
| Interpersonal/social relationship | 3,19 | 50 | 50 | 0 |
| Autonomy/Control/Dependence | 3,03 | 77,78 | 22,22 | 0 |
| Adaptation/Coping | 2,98 | 66,67 | 33,33 | 0 |
| Social activities | 2,95 | 44,44 | 44,44 | 11,11 |
| Enthusiasm/Pleasure | 2,69 | 61,11 | 38,89 | 0 |
| Usual/Daily activities | 2,63 | 38,89 | 50 | 11,11 |
| Dexterity/Handling | 2,49 | 44,44 | 55,56 | 0 |
| Self-care | 2,42 | 66,67 | 27,78 | 5,56 |
| Physical discomfort/Pain | 2,41 | 44,44 | 50 | 5,56 |
| Sadness/Depression | 2,41 | 61,11 | 33,33 | 5,56 |
| Intimacy/Sexuality | 2,41 | 27,78 | 61,11 | 11,11 |
| Anxiety/Distress | 2,03 | 50 | 44,44 | 5,56 |
| Suicidal ideations | 2,02 | 38,89 | 38,89 | 22,22 |
| Social inclusion/Connectedness | 1,89 | 16,67 | 72,22 | 11,11 |
| Terror/Panic/Fear | 1,74 | 38,89 | 44,44 | 16,67 |
| Fertility | 1,74 | 33,33 | 44,44 | 22,22 |
| Integration into the community | 1,68 | 27,78 | 61,11 | 11,11 |
| Feeling of being a burden to others | 1,65 | 27,78 | 55,56 | 16,67 |
| Loneliness | 1,63 | 33,33 | 50 | 16,67 |
| Calm/Agitation/Irritability | 1,53 | 33,33 | 55,56 | 11,11 |
| Anger | 1,40 | 27,78 | 55,56 | 16,67 |
| Appearance | 1,35 | 11,11 | 66,67 | 22,22 |
| Humiliation/Shame | 1,34 | 27,78 | 44,44 | 27,78 |
| Others (10 categories suggested): | 2,95 |  |  |  |
| Being accepted and understood / Being meaningful/respected |  |  |  |  |
| Access to resources (money, education, employment, etc.) |  |  |  |  |
| Ability to trust / respect |  |  |  |  |
| Spirituality |  |  |  |  |
| Introspection and self-knowledge |  |  |  |  |
| Self-acceptance and self-respect |  |  |  |  |
| Sense of achievement and purpose |  |  |  |  |
| Freedom and self-determination |  |  |  |  |
| Psychological distress and stress |  |  |  |  |
| Citizen role |  |  |  |  |

**A2:** Comparison between the first proposed instrument (round 2) and the derived one through the Delphi procedure (round 4) (original versions were in French and this translation does not correspond to the final 13-MD)

| First proposed instrument. | Instrument derived through the Delphi procedure (15-D) |
| --- | --- |
| **Body Functioning**   - I have no difficulty in breathing, feeding, or urinating/defecating - I have some difficulty in breathing, feeding, or urinating/defecating - I have difficulty in breathing, feeding, or urinating/defecating - I have great difficulty in breathing, feeding, or urinating/defecating - I have very great difficulty in breathing, feeding, or urinating/defecating - I am in physical distress and/or need assistance to urinate/defecate | \| **Body Functioning** \| \| \| \| \| --- \| --- \| --- \| --- \| \|  \| **Breathing** \| **Feeding** \| **Eliminating** (urine and feces) \| \| I have no difficulty in ... \|  \|  \|  \| \| I have very little difficulty in... \|  \|  \|  \| \| I have a little difficulty in ... \|  \|  \|  \| \| I have difficulty in… \|  \|  \|  \| \| I have great difficulty in ... \|  \|  \|  \| \| I have very great difficulty in ... \|  \|  \|  \| \| I need assistance with ... \|  \|  \|  \| |
| **Cognition (ability to think, remember, etc.), senses (vision, hearing, etc.) and language (speaking)**   - I have no cognitive problems and no difficulty with senses and language - I have no cognitive problems but have difficulty with some senses and language - I have a mild cognitive problem and/or significant difficulty with some senses - I have a significant cognitive problem and/or great difficulty with some senses and language - I have a very significant cognitive problem and difficulty with some senses and language - I have a very significant cognitive problem and great difficulty with some senses and language | \| **Cognition, senses, and language** \| \| \| \| \| --- \| --- \| --- \| --- \| \|  \| **my cognition** (thinking, remembering, concentrating, etc.) \| **certain senses** (vision, hearing, etc.) \| **language** (speaking, making myself understood, etc.) \| \| I have no difficulty with ... \|  \|  \|  \| \| I have very little difficulty with... \|  \|  \|  \| \| I have some difficulty with ... \|  \|  \|  \| \| I have difficulty with… \|  \|  \|  \| \| I have great difficulty with ... \|  \|  \|  \| \| I have very great difficulty with ... \|  \|  \|  \| |
| **Sleep and energy**   - I have no sleep problems and I have a lot of energy - I have no sleep problems, but I lack energy - I have a little trouble sleeping and I lack energy - I have trouble sleeping and lack energy - I have trouble sleeping and have very little energy - I have great difficulty sleeping and/or I am exhausted and drained | \| **Sleep and energy** \| \| \| \| \| --- \| --- \| --- \| --- \| \|  \| **Sleep** \|  \| **Energy** \| \| I don't have a problem with ... \|  \| I don't lack… \|  \| \| I have a very slight problem with ... \|  \| I have a very slight lack of ... \|  \| \| I have a slight problem with ... \|  \| I have a slight lack of ... \|  \| \| I have problems with… \|  \| I have a lack of… \|  \| \| I have a significant problem with ... \|  \| I have a very small ... \|  \| \| I have a very important problem of ... \|  \| I am exhausted and without… \|  \| |
| **Self-esteem and self-acceptance**   - I don't have any confidence or self-esteem problems - I have no confidence or self-esteem problems, but I have difficulty accepting myself - I have some lack of confidence or self-esteem and have difficulty accepting myself - I lack a lot of confidence or self-esteem and have difficulty accepting myself - I have very little confidence or self-esteem and have difficulty accepting myself - I have a lot of difficulty accepting and respecting myself | \| **Self-esteem and self-acceptance** \| \| \| \| \| --- \| --- \| --- \| --- \| \|  \| **confidence or self-esteem** \|  \| **accept myself** \| \| I have no problem with ... \|  \| I have no difficulty to ... \|  \| \| I have a very slight problem with ... \|  \| I have very little difficulty to ... \|  \| \| I have a slight problem with ... \|  \| I have a little difficulty to ... \|  \| \| I have problems with… \|  \| I have difficulty to … \|  \| \| I have a major problem with ... \|  \| I have great difficulty to... \|  \| \| I have a very big problem with ... \|  \| I have a very big problem to... \|  \| \| I feel like a loser, and I can't measure up. I don't have any ... \|  \| I do not deserve any consideration. It is impossible for me to ... \|  \| |
| **Pain and physical discomfort**   - I have no pain or physical discomfort - I have no pain, but I have physical discomfort - I have mild pain and physical discomfort - I have moderate pain and physical discomfort - I have severe pain and a lot of physical discomfort - I have very severe pain and a lot of physical discomfort | \| **Pain and physical discomfort** \| \| \| \| \| --- \| --- \| --- \| --- \| \|  \| **Pain** \|  \| **Discomfort** \| \| I have no ... \|  \| I have no ... \|  \| \| I have a very mild ... \|  \| I have a very mild ... \|  \| \| I have a mild ... \|  \| I have a mild ... \|  \| \| I have… \|  \| I have… \|  \| \| I have an intense ... \|  \| I have a lot of ... \|  \| \| I have a very intense ... \|  \| I have huge … \|  \| |
| **Mobility and physical ability**   - I have no difficulty performing demanding activities (e.g., running, lifting) - I have some difficulty with strenuous activities (e.g., running, lifting) - I have some difficulty with moderate activities (e.g. walking, golfing) - I have great difficulty with moderate activities (e.g., walking, golfing) - I have great difficulty with bathing, dressing or feeding myself - I am unable to bathe, dress or feed myself | \| **Mobility and physical disability** \| \| \| \| \| --- \| --- \| --- \| --- \| \|  \| **perform strenuous activities** (e.g., running, lifting, etc.) \| **perform moderate activities** (walking, climbing stairs, playing golf, etc.) \| **bathe, dress or feed myself** \| \| I have no difficulty to... \|  \|  \|  \| \| I have very little difficulty to... \|  \|  \|  \| \| I have some difficulty to ... \|  \|  \|  \| \| I have difficulty to \|  \|  \|  \| \| I have great difficulty to ... \|  \|  \|  \| \| I have very great difficulty to ... \|  \|  \|  \| \| I am not able to... \|  \|  \|  \| |
| **Daily activities and work**   - I have no difficulty performing routine activities (cooking, cleaning, shopping, etc.) - I have no difficulty performing routine activities (cooking, cleaning, errands, etc.) but I have difficulty performing things at work or school - I have difficulty with everyday activities (cooking, cleaning, errands, etc.) and with things at work or school - I have a lot of difficulty with everyday activities (cooking, cleaning, errands, etc.) and with getting things done at work or school - I am not able to find and keep a job or finish school - I am unable to perform any routine activities (cooking, cleaning, errands, etc.) | \| **Daily activities and work** \| \| \| \| --- \| --- \| --- \| \|  \| **do everyday activities** (cooking, cleaning, shopping, etc.) \| **perform work- or study-related activities** (organise my tasks, hand in my work on time, etc.) \| \| I have no difficulty to ... \|  \|  \| \| I have very little difficulty to ... \|  \|  \| \| I have some difficulty to ... \|  \|  \| \| I have difficulty to… \|  \|  \| \| I have great difficulty to ... \|  \|  \| \| I have very great difficulty to ... \|  \|  \| \| I am not able to... \|  \|  \| |
| **Social and leisure activities**   - I do as many social and leisure activities as I want - I do almost as many social and leisure activities as I want - I do less social and leisure activities than I want to - I do far less social and leisure activities than I want to - I never do as many social and leisure activities as I want - I can't do any social or leisure activities even though I want to | \| **Social and leisure activities** \| \| \| \| \| --- \| --- \| --- \| --- \| \|  \| **social** \| **leisure** \|  \| \| I do as many activities ... \|  \|  \| as I want \| \| I do almost as many activities … \|  \|  \| as I want \| \| I do fewer activities ... \|  \|  \| than I would like \| \| I do far fewer activities ... \|  \|  \| than I would like \| \| I do infinitely fewer activities ... \|  \|  \| than I would like \| \| I cannot do any activities ... \|  \|  \| despite my desire to do so \| |
| **Social and interpersonal relationships**   - I am accepted and listened to by those around me and we share affection and respect - I am accepted and listened to by those around me but I lack affection and respect - I am little accepted and listened to by my entourage, but we share affection and respect - I am little accepted and listened to by my entourage and I lack affection and respect - I cannot maintain quality relationships with those around me - I am excluded by my environment, and I feel very isolated | \| **Social and interpersonal relationships** \| \| \| \| \| \| --- \| --- \| --- \| --- \| --- \| \|  \| **accepted and listened to by my circle** \|  \| **affection and support** \| \| I am feeling fully ... \|  \| I receive a lot of ... \|  \| \| I am feeling fairly well ... \|  \| I receive enough ... \|  \| \| I am feeling little ... \|  \| I receive little ... \|  \| \| I am feeling very little ... \|  \| I receive very little ... \|  \| \| I am not feeling... \|  \| I do not receive any ... \|  \| \| I am feeling excluded and/or harassed \|  \| I feel isolated and/or vulnerable \|  \| |
| **Citizenship and social inclusion**   - I am fully engaged in my role as a citizen and highly integrated into society - I am as engaged as possible in my role as a citizen and am very integrated into society - I am as engaged as possible in my role as a citizen and fairly well integrated into society - I am not very involved in my role as a citizen but am fairly well integrated into society - I am not very involved in my role as a citizen and am not well integrated into society - I am very little involved in my role as a citizen and very little integrated into society | \| **Citizenship and social inclusion** \| \| \| \| --- \| --- \| --- \| \|  \| **engaged in my role as a citizen (contributing to my community, family, work, etc.)** \| **integrated into society** \| \| I am fully ... \|  \|  \| \| I am quite well ... \|  \|  \| \| I am sufficiently… \|  \|  \| \| I am little ... \|  \|  \| \| I am very little ... \|  \|  \| \| I am not ... \|  \|  \| |
| **Autonomy**   - I feel fully autonomous and able to self-determine my life - I feel autonomous but I sometimes have difficulty coping with unexpected situations - I feel little autonomy and often have difficulty coping with unexpected situations - I feel very little autonomy and I feel I have little control over my life - I feel trapped and have very little control over my life - I feel trapped and feel totally dependent on others | \| **Autonomy and adaptation** \| \| \| \| \| --- \| --- \| --- \| --- \| \|  \| **autonomous and free to make my own choices** \|  \| **face unexpected situations** \| \| I am totally ... \|  \| I have no difficulty to ... \|  \| \| I am very… \|  \| I have little difficulty to ... \|  \| \| I am somewhat ... \|  \| I have difficulty to… \|  \| \| I have little ... \|  \| I have great difficulty to ... \|  \| \| I have very little ... \|  \| I have very great difficulty to ... \|  \| \| I am not ... \|  \| I am not able to ... \|  \| |
| **Anxiety and depression**   - I am not depressed, sad or anxious at all - I am somewhat depressed, sad, or anxious - I am somewhat depressed, sad, or anxious - I am very depressed, sad, or anxious - I am in great psychological pain - I am in great psychological distress and have suicidal thoughts | \| **Depression, anxiety, and anger** \| \| \| \| \| --- \| --- \| --- \| --- \| \|  \| **sad or depressed** \| **anxious or stressed** \| **angry or irritated** \| \| I am never ... \|  \|  \|  \| \| I am rarely… \|  \|  \|  \| \| I am sometimes... \|  \|  \|  \| \| I am often ... \|  \|  \|  \| \| I am very often ... \|  \|  \|  \| \| I am always ... \|  \|  \|  \| |
|  | \| **Insecurity and fear** \| \| \| \| --- \| --- \| --- \| \|  \| **In safety** \| **Scared or worried** \| \| I never feel ... \|  \|  \| \| I rarely feel ... \|  \|  \| \| I sometimes feel ... \|  \|  \| \| I often feel ... \|  \|  \| \| I very often feel... \|  \|  \| \| I always feel ... \|  \|  \| |
| **Well-being**   - My well-being is very high because I often feel fulfilled, useful, and satisfied with my life - My well-being is high because I often feel fulfilled, useful, and satisfied with my life - My well-being is fairly high because I often feel fulfilled, useful and satisfied with my life - My well-being is low because I do not feel fulfilled, useful, and satisfied with my life very often - My well-being is very low because I rarely feel fulfilled, useful and satisfied with my life - My well-being is extremely low because I never feel fulfilled, useful and satisfied with my life | \| **Well-being** \| \| \| \| \| --- \| --- \| --- \| --- \| \|  \| **fulfilled** \| **useful** \| **satisfied with my life** \| \| I always feel ... \|  \|  \|  \| \| I feel very often ... \|  \|  \|  \| \| I often feel ... \|  \|  \|  \| \| I feel sometimes ... \|  \|  \|  \| \| I rarely feel ... \|  \|  \|  \| \| I never feel ... \|  \|  \|  \| |
| **Sexuality and intimacy**   - I am very satisfied with my sex life and my level of intimacy with my partner - I am satisfied with my sex life and my level of intimacy with my partner - I am not very satisfied with my sex life and my level of intimacy with my partner - I am dissatisfied with my sex life and level of intimacy with my partner - I am very dissatisfied with my sex life and level of intimacy with my partner - I am very dissatisfied because I do not have a shared sex life and intimacy | \| **Sexuality and intimacy** \| \| \| \| \| --- \| --- \| --- \| --- \| \|  \| **my sex life** \| **my level of intimacy with my partner** \| **my sexual identity** \| \| I am totally satisfied with ... \|  \|  \|  \| \| I am very satisfied with… \|  \|  \|  \| \| I am somewhat satisfied with ... \|  \|  \|  \| \| I am little satisfied with ... \|  \|  \|  \| \| I am very little satisfied with ... \|  \|  \|  \| \| I am dissatisfied with ... \|  \|  \|  \| \| I am very dissatisfied with ... \|  \|  \|  \| |

**A3:** Model 1 results after the CFA structural equation modeling estimation.

**
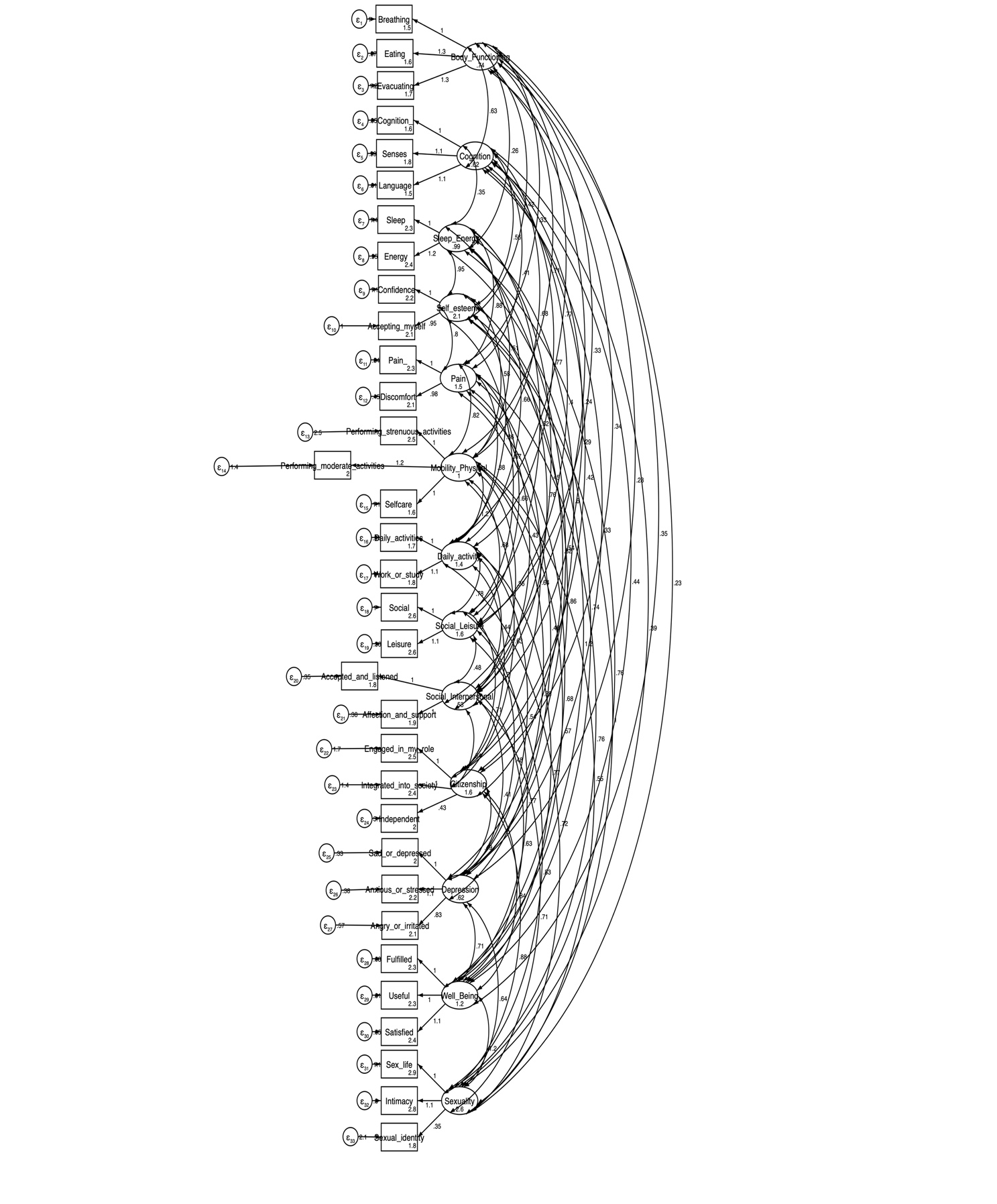
**

**A4:** The 13-MD (translated from French by two independent professionals)

***Please complete this health-related quality of life questionnaire, considering your condition during the current week:***

| **Body Functioning** | | | |
| --- | --- | --- | --- |
|  | **breathing** | **eating** | **evacuating** (urine and fecal matter) |
| I have no difficulty with ... |  |  |  |
| I have very little difficulty with... |  |  |  |
| I have a little difficulty with ... |  |  |  |
| I have difficulty with… |  |  |  |
| I have a lot of difficulty with ... |  |  |  |
| I have tremendous difficulty with ... |  |  |  |
| I need assistance with ... |  |  |  |

| **Cognition, senses and language** | | | | |
| --- | --- | --- | --- | --- |
|  | **my cognition** (thinking, remembering, concentrating, etc.) | **some of my senses** (sight, hearing, etc.) | **language** (speaking, making myself understood, etc.) |  |
| I have no difficulty with ... |  |  |  |  |
| I have very little difficulty with... |  |  |  |  |
| I have a little difficulty with ... |  |  |  |  |
| I have difficulty with… |  |  |  |  |
| I have a lot of difficulty with ... |  |  |  |  |
| I have tremendous difficulty with ... |  |  |  |  |

| **Sleep and energy** | | | | |
| --- | --- | --- | --- | --- |
|  | **sleep** |  | **energy** |  |
| I have no problems with ... |  | I do not lack any… |  |  |
| I have very slight problems with ... |  | I lack very little of ... |  |  |
| I have slight problems with ... |  | I lack a little of ... |  |  |
| I have problems with… |  | I lack of… |  |  |
| I have a lot of problems with ... |  | I have very little ... |  |  |
| I have tremendous problems with ... |  | I am exhausted and without… |  |  |

| **Self-esteem and self-acceptance** | | | | |
| --- | --- | --- | --- | --- |
|  | **confidence or self-esteem** |  | **accepting myself** |  |
| I have no problems with ... |  | I have no difficulty ... |  |  |
| I have very slight problems with ... |  | I have very little difficulty ... |  |  |
| I have slight problems with ... |  | I have a little difficulty ... |  |  |
| I have problems with… |  | I have difficulty … |  |  |
| I have a lot of problems with ... |  | I have a lot of difficulty... |  |  |
| I have tremendous problems with ... |  | I have tremendous difficulty... |  |  |
| I feel like a loser and never fit in. I have no ... |  | I do not deserve any consideration. I am completely incapable of ... |  |  |

| **Physical pain and discomfort** | | | |
| --- | --- | --- | --- |
|  | **pain** |  | **discomfort** |
| I have no ... |  | I feel no ... |  |
| I have very mild ... |  | I feel very mild ... |  |
| I have mild ... |  | I feel mild ... |  |
| I have… |  | I feel… |  |
| I have intense ... |  | I feel a lot of ... |  |
| I have very intense ... |  | I feel a very significant … |  |

| **Mobility and physical disability** | | | |
| --- | --- | --- | --- |
|  | **performing strenuous activities** (running, lifting weights, etc.) | **performing moderate activities** (walking, climbing stairs, playing golf, etc.) | **taking a bath, getting dressed or feeding myself** |
| I have no difficulty ... |  |  |  |
| I have very little difficulty ... |  |  |  |
| I have a little difficulty ... |  |  |  |
| I have difficulty |  |  |  |
| I have a lot of difficulty ... |  |  |  |
| I have tremendous difficulty ... |  |  |  |
| I am incapable of ... |  |  |  |

| **Daily activities and work** | | |
| --- | --- | --- |
|  | **accomplishing daily activities** (cooking, doing household chores, running errands, etc.) | **accomplishing work- or school-related activities** (planning my tasks, getting to work on time, etc.) |
| I have no difficulty ... |  |  |
| I have very little difficulty ... |  |  |
| I have a little difficulty ... |  |  |
| I have difficulty … |  |  |
| I have a lot of difficulty ... |  |  |
| I have tremendous difficulty ... |  |  |
| I am incapable of ... |  |  |

| **Social and leisure activities** | | | |
| --- | --- | --- | --- |
|  | **social** | **leisure** |  |
| I participate in as many ... |  |  | activities as I like |
| I participate in nearly as many … |  |  | activities as I would like |
| I participate in fewer ... |  |  | activities than I would like |
| I participate in significantly fewer ... |  |  | activities than I would like |
| I participate in infinitely fewer ... |  |  | activities than I would like |
| I cannot participate in any ... |  |  | activity, despite my desire to do so |

| **Social and interpersonal relationships** | | | | | | | |  |
| --- | --- | --- | --- | --- | --- | --- | --- | --- |
|  | | **accepted and listened by those around me** | |  | | **affection and support** | |  |
| I feel fully ... | |  | | I get a lot of ... | |  | |  |
| I feel kind of ... | |  | | I get enough ... | |  | |  |
| I feel only a little ... | |  | | I get a little ... | |  | |  |
| I feel very little ... | |  | | I get very little ... | |  | |  |
| I feel excluded and not at all | |  | | I get no … | |  | |  |
| **Citizenship, inclusion and autonomy** | | | | |  | |  | |
|  | **engaged in my role as a citizen (contributing to my community, family, work, etc.)** | | **integrated into society** | |  | | **autonomous and free to make my own choices** | |
| I am fully ... |  | |  | | I am completely ... | |  | |
| I am rather well ... |  | |  | | I am very… | |  | |
| I am sufficiently… |  | |  | | I am rather ... | |  | |
| I am little ... |  | |  | | I am little ... | |  | |
| I am very little ... |  | |  | | I am very little ... | |  | |
| I am not ... |  | |  | | I am not ... | |  | |

| **Depression, anxiety and anger** | | | |
| --- | --- | --- | --- |
|  | **sad or depressed** | **anxious or stressed** | **angry or irritated** |
| I am never ... |  |  |  |
| I am rarely… |  |  |  |
| I am occasionally ... |  |  |  |
| I am often ... |  |  |  |
| I am very often ... |  |  |  |
| I am always ... |  |  |  |

| **Well-being** | | | |
| --- | --- | --- | --- |
|  | **fulfilled** | **useful** | **satisfied with my life** |
| I always feel ... |  |  |  |
| I feel very often ... |  |  |  |
| I often feel ... |  |  |  |
| I occasionally feel ... |  |  |  |
| I rarely feel ... |  |  |  |
| I never feel ... |  |  |  |

| **Sexuality and intimacy** | | | | |
| --- | --- | --- | --- | --- |
|  | **my sex life** | **my level of intimacy with my partner** | **my sexual identity** |  |
| I am totally satisfied with ... |  |  |  |  |
| I am very satisfied with… |  |  |  |  |
| I am rather satisfied with ... |  |  |  |  |
| I am little satisfied with ... |  |  |  |  |
| I am very little satisfied with ... |  |  |  |  |
| I am dissatisfied with ... |  |  |  |  |
| I am very dissatisfied with ... |  |  |  |  |

**A5:** The 13-MD in French

***On vous demande de compléter ce questionnaire de qualité de vie reliée à la santé en considérant votre situation pour la semaine en cours :***

| **Fonctionnement du corps** | | | | |
| --- | --- | --- | --- | --- |
|  | **respirer** | **m’alimenter** | **éliminer** (urine et matières fécales) |  |
| Je n’ai **aucune** difficulté à … |  |  |  |  |
| J’ai **très peu** de difficulté à … |  |  |  |  |
| J’ai un **peu** de difficulté à … |  |  |  |  |
| J’ai de la difficulté à … |  |  |  |  |
| J’ai de **grandes** difficultés à … |  |  |  |  |
| J’ai de **très grandes** difficultés à … |  |  |  |  |
| J’ai **besoin d’assistance** pour … |  |  |  |  |

| **Cognition, sens et langage** | | | |
| --- | --- | --- | --- |
|  | **ma cognition** (réfléchir, me souvenir, me concentrer, etc.) | **certains sens** (vision, ouïe, etc.) | **le langage** (parler, me faire comprendre, etc.) |
| Je n’ai **aucune** difficulté avec … |  |  |  |
| J’ai **très peu** de difficulté avec … |  |  |  |
| J’ai un **peu** de difficulté avec … |  |  |  |
| J’ai de la difficulté avec … |  |  |  |
| J’ai de **grandes** difficultés avec … |  |  |  |
| J’ai de **très grandes** difficultés avec … |  |  |  |

| **Sommeil et énergie** | | | | |
| --- | --- | --- | --- | --- |
|  | **sommeil** |  | **énergie** |  |
| Je n’ai **aucun** problème de … |  | Je ne manque **pas** d’… |  |  |
| J’ai un **très léger** problème de … |  | Je manque **très légèrement** d’… |  |  |
| J’ai un **léger** problème de … |  | Je manque **légèrement** d’… |  |  |
| J’ai un problème de … |  | Je manque d’… |  |  |
| J’ai un **important** problème de … |  | J’ai **très peu** d’… |  |  |
| J’ai un **très important** problème de … |  | Je suis **épuisé** et **sans…** |  |  |

| **Estime et acceptation de soi** | | | |
| --- | --- | --- | --- |
|  | **confiance ou estime de moi** |  | **m’accepter** |
| Je n’ai **aucun** problème de … |  | Je n’ai **aucune** difficulté à … |  |
| J’ai un **très léger** problème de … |  | J’ai **très peu** de difficulté à … |  |
| J’ai un **léger** problème de … |  | J’ai un **peu** de difficulté à … |  |
| J’ai un problème de … |  | J’ai de la difficulté à … |  |
| J’ai un **important** problème de … |  | J’ai de **grandes** difficultés à … |  |
| J’ai un **très important** problème de … |  | J’ai de **très grandes** difficultés à … |  |
| Je me sens nul et jamais à la hauteur. Je n’ai **aucune** … |  | Je ne mérite aucune considération. Il m’est **impossible** de … |  |

| **Douleur et inconfort physique** | | | |
| --- | --- | --- | --- |
|  | **douleur(s)** |  | **inconfort** |
| Je n’ai **aucune** … |  | Je n’ai **aucun** … |  |
| J’ai de **très légères** … |  | J’ai un **très léger** … |  |
| J’ai de **légères** … |  | J’ai un **léger** … |  |
| J’ai des … |  | J’ai de l’… |  |
| J’ai d’**intenses** … |  | J’ai **beaucoup** d’… |  |
| J’ai de **très intenses** … |  | J’ai **énormément** d’… |  |

| **Mobilité et incapacité physique** | | | | |
| --- | --- | --- | --- | --- |
|  | **accomplir des activités intenses** (courir, soulever un poids, etc.) | **accomplir des activités modérées** (marcher, monter des marches, jouer au golf, etc.) | **prendre un bain, m’habiller ou me nourrir seul** |  |
| Je n’ai **aucune** difficulté à … |  |  |  |  |
| J’ai **très peu** de difficulté à … |  |  |  |  |
| J’ai un **peu** de difficulté à … |  |  |  |  |
| J’ai de la difficulté à … |  |  |  |  |
| J’ai de **grandes** difficultés à … |  |  |  |  |
| J’ai de **très grandes** difficultés à … |  |  |  |  |
| Je ne suis **pas capable** d’… |  |  |  |  |

| **Activités courantes et travail** | | |
| --- | --- | --- |
|  | **accomplir des activités courantes** (cuisiner, faire le ménage, les commissions, etc.) | **accomplir des activités liées au travail ou aux études** (organiser mes tâches, rendre mon travail à temps, etc.) |
| Je n’ai **aucune** difficulté à … |  |  |
| J’ai **très peu** de difficulté à … |  |  |
| J’ai un **peu** de difficulté à … |  |  |
| J’ai de la difficulté à … |  |  |
| J’ai de **grandes** difficultés à … |  |  |
| J’ai de **très grandes** difficultés à … |  |  |
| Je ne suis **pas capable** d’… |  |  |

| **Activités sociales et loisirs** | | | | |
| --- | --- | --- | --- | --- |
|  | **sociales** | **loisirs** |  |  |
| Je pratique **autant** d’activités … |  |  | que je le souhaite |  |
| Je pratique **presque autant** d’activités … |  |  | que je le souhaite |  |
| Je pratique **moins** d’activités … |  |  | que je le souhaite |  |
| Je pratique **beaucoup moins** d’activités … |  |  | que je le souhaite |  |
| Je pratique **infiniment moins** d’activités … |  |  | que je le souhaite |  |
| Je ne peux pratiquer **aucune** activité … |  |  | malgré mon désir d’en faire |  |

| **Relations sociales et interpersonnelles** | | | | |
| --- | --- | --- | --- | --- |
|  | **accepté et écouté** par mon entourage |  | **affection et de soutien** |  |
| Je me sens **pleinement** … |  | Je reçois **beaucoup** d’… |  |  |
| Je me sens **plutôt bien** … |  | Je reçois **suffisamment** d’… |  |  |
| Je me sens **peu** … |  | Je reçois **peu** d’… |  |  |
| Je me sens **très peu** … |  | Je reçois **très peu** d’… |  |  |
| Je me sens exclu et **pas du tout** … |  | Je ne reçois **aucune** … |  |  |

| **Citoyenneté, inclusion et autonomie** | | | | | |
| --- | --- | --- | --- | --- | --- |
|  | **engagé dans mon rôle de citoyen** (en contribuant à ma communauté, ma famille ou mon travail, etc.) | **intégré dans la société** |  | **autonome et libre de faire mes propres choix** |  |
| Je suis **pleinement** … |  |  | Je suis **totalement** … |  |  |
| Je suis **plutôt bien** … |  |  | Je suis **très** … |  |  |
| Je suis **suffisamment** … |  |  | Je suis **plutôt** … |  |  |
| Je suis **peu** … |  |  | Je suis **peu** … |  |  |
| Je suis **très peu** … |  |  | Je suis **très peu** … |  |  |
| Je ne suis **pas** … |  |  | Je ne suis **pas** … |  |  |

| **Dépression, anxiété et colère** | | | | |
| --- | --- | --- | --- | --- |
|  | **triste ou déprimé** | **anxieux ou stressé** | **en colère ou irrité** |  |
| Je ne suis **jamais** … |  |  |  |  |
| Je suis **rarement** … |  |  |  |  |
| Je suis **parfois** … |  |  |  |  |
| Je suis **souvent** … |  |  |  |  |
| Je suis **très souvent** … |  |  |  |  |
| Je suis **toujours** … |  |  |  |  |

| **Bien-être** | | | |
| --- | --- | --- | --- |
|  | **épanoui** | **utile** | **satisfait de ma vie** |
| Je me sens **toujours** … |  |  |  |
| Je me sens **très souvent** … |  |  |  |
| Je me sens **souvent** … |  |  |  |
| Je me sens **parfois** … |  |  |  |
| Je me sens **rarement** … |  |  |  |
| Je ne me sens **jamais** … |  |  |  |

| **Sexualité et intimité** | | | | |
| --- | --- | --- | --- | --- |
|  | **ma vie sexuelle** | **mon niveau d’intimité avec mon/ma partenaire** | **mon identité sexuelle** |  |
| Je suis **totalement satisfait** de … |  |  |  |  |
| Je suis **très satisfait** de … |  |  |  |  |
| Je suis **plutôt satisfait** de … |  |  |  |  |
| Je suis **peu satisfait** de … |  |  |  |  |
| Je suis **très peu satisfait** de … |  |  |  |  |
| Je suis **insatisfait** de … |  |  |  |  |
| Je suis **très insatisfait** de … |  |  |  |  |
